# Real‑world safety and efficacy of biological agents in inflammatory bowel disease: a one-year post-marketing pharmacovigilance observational study in the Calabria region

**DOI:** 10.1007/s43440-025-00774-x

**Published:** 2025-08-19

**Authors:** Antonio Fabiano, Caterina De Sarro, Domenico Frajia, Francesca Bosco, Lorenza Guarnieri, Stefano Ruga, Stefano Rodinò, Ladislava Sebkova, Enrico Ciliberto, Isidoro Buoncompagni, Laura Costantino, Antonio Leo, Gianmarco Marcianò, Vincenzo Rania, Rita Citraro, Ashour Michael, Giovambattista De Sarro

**Affiliations:** 1 Department of Health Sciences, Magna Graecia University of Catanzaro, Catanzaro, 88100 Italy; 2 Science of Health Department, School of Medicine, System and Applied Pharmacology@University Magna Grecia, Magna Graecia University of Catanzaro, Catanzaro, 88100 Italy; 3 Gastroenterology Unit, Pugliese-Ciaccio Hospital, Catanzaro, Italy; 4 Gastroenterology Unit, San Giovanni di Dio Hospital, Crotone, Italy

**Keywords:** Inflammatory bowel diseases (IBDs), Biologics, Monoclonal antibodies, Post-marketing surveillance, Pharmacovigilance, Adverse events (AEs)

## Abstract

**Background:**

The use of immune-modifying biological agents has markedly changed the clinical course and the management of inflammatory bowel diseases (IBDs). Active post-marketing surveillance programs are essential for the early recognition of both expected and unexpected adverse events (AEs), providing a powerful tool for better defining the safety profiles of biologics in a real-world setting.

**Methods:**

Patients diagnosed with IBDs and treated with biologic drugs at two gastroenterology units in Calabria, Italy, were monitored during the period from 2023 to 2024. AEs and drug switches or swaps were recorded. The primary objective was to assess the safety profile of biological therapies in real-world practice, as measured by the occurrence of AEs. Secondary objectives focused on assessing treatment effectiveness by monitoring rates of therapeutic ineffectiveness and rigorously analyzing necessary modifications to therapy (swaps/switches) in response to treatment failure.

**Results:**

A total of 214 patients were enrolled, including 85 with Crohn’s disease (CD) and 120 with ulcerative colitis (UC). Among biologics, vedolizumab (VDZ) was the most prescribed drug (50.3%), followed by ustekinumab (UST, 33.6%). Among biosimilars, infliximab (IFX) was the most administered (70%), followed by adalimumab (ADA) (63.3%). 96 patients experienced AEs, though no serious adverse events (SAEs) were reported. The highest number of AEs was reported with VDZ (*n* = 31; 32.3%), followed by IFX (*n* = 22, 23.0%), ADA and UST (*n* = 17, 17.7%), and golimumab (GOL) (*n* = 7; 7.3%). The biological drugs associated with the fewest AEs were upadacitinib (UPA) and tofacitinib (TFC) (*n* = 1; 1.0%).

**Conclusions:**

This study confirms the importance of pharmacovigilance in monitoring the safety of biologics in IBDs. The results offer useful insights into the actual use of monoclonals in gastroenterology and support more targeted prescribing.

**Clinical trial number:**

Not applicable.

## Introduction

Inflammatory bowel diseases (IBDs), including ulcerative colitis (UC) and Crohn’s disease (CD), are chronic inflammatory conditions of the gastrointestinal (GI) tract driven by an abnormal immune response to gut microbiota [1]. Though distinct, UC and CD often share clinical features and typically arise in young adults. Their chronic, relapsing nature leads to complications, reduced quality of life, and a higher prevalence of extra-intestinal manifestations affecting the musculoskeletal, dermatological, hepatic, endocrine, and cardiovascular systems [2–4]. Approximately 0.2% of the European population is affected by IBDs, with Italy reporting ~ 260,000 cases [5]. The clinical and socioeconomic burden is substantial, including increased healthcare costs, work disability, and psychological distress.

IBDs pathogenesis involves complex interactions between genetic, environmental, microbial, and immune factors. A central role is played by mucosal immunity, with T helper 1 (Th1)-mediated responses predominating in CD and atypical T helper 2 (Th2) lymphocytes profiles in UC. T helper 17 (Th17) lymphocytes, epithelial barrier dysfunction, dysbiosis, and impaired autophagy are also implicated [6–8].

Conventional treatments include mesalazine (MSZ), corticosteroids (CCSs), antibiotics, and immunosuppressants. The introduction of biologics, beginning with anti-tumor necrosis factor (TNF) agents like infliximab (IFX), marked a turning point in IBDs management. Today, multiple biologics are approved in Italy: anti-TNF agents (IFX, adalimumab (ADA), golimumab (GOL)), anti-integrins (vedolizumab (VDZ)), and anti-interleukin-12/23 (IL-12/23) agents (ustekinumab (UST)) [9]. These therapies have improved clinical outcomes; however, primary non-response, loss of efficacy, adverse events (AEs), serious adverse events (SAEs), and high costs remain significant challenges [10].

Treatment selection is complex and requires balancing efficacy, safety, and cost. Real-world data and post-marketing pharmacovigilance are crucial to inform clinical decisions [11, 12]. This observational study investigates the safety and effectiveness of biologics in IBDs patients across two gastroenterology units in Calabria, Italy, as part of the Calabria Biologics Pharmacovigilance Program (CBPP).

## Materials and methods

In this study, data from patients who met the inclusion criteria and were treated with a biological drug for one year (2023–2024) in the gastroenterology units of two hospitals in Calabria were examined. Clinical pharmacology specialists developed a database in compliance with Good Clinical Practice guidelines. They collected demographic and clinical data, as well as information on therapies, treatment failures, drug switches, and AEs recorded during the study period. In this study, we define a switch as the replacement of a biological agent with another of the same class (i.e., a biosimilar), and a swap as the replacement of a biological drug with one of a different class. To preserve patient privacy, an encrypted code was used for each patient, in accordance with the Declaration of Helsinki (1964) and its subsequent revisions. The study protocol was accepted by the Calabria Regional Ethics Committee, under protocol number 278/2015 and protocol number 387 of 19/11/2020.

### Study design

This two-center real-world pharmacovigilance study aims to enhance the continuous monitoring of the safety of biological therapies in real practice. The primary objective was to assess the safety profile of biological therapies in real-world practice, as measured by the occurrence of AEs. Secondary objectives included evaluating treatment effectiveness by assessing rates of therapeutic ineffectiveness and analyzing therapy modifications (swaps/switches) in response to treatment failure. This is a retrospective observational study based on real-world data collected from medical records and routine clinical follow-up. As previously described [13], the study includes an initial phase of training physicians on pharmacovigilance data and the correct documentation of AEs. All patients treated with a biological agent at 2 hospital gastroenterology centers (the O.U. of Gastroenterology of the university hospital company ‘Renato-Dulbecco’ (hospital pole of Pugliese-Ciaccio) Calabria, Italy, and the O.U. of Gastroenterology of the civil hospital ‘San Giovanni di Dio’ of Crotone, Calabria, Italy) between 2 November 2023 and 1 November 2024 were screened for eligibility.

### Inclusion criteria

In this study, 214 patients were enrolled, of which 109 were naïve patients and 105 were treated with biological agents. All patients met the inclusion criteria: age > 18 years; diagnosis of moderate to severe CD or UC, and treatment with a biological agent. All patients provided informed consent at admission. The start of biological therapy, whether in naive or previously treated patients, was defined as the index date of the study. A hybrid system combining telephone calls from the pharmacologist with routine specialist visits, as previously reported [13], was employed to monitor side effects throughout the follow-up period, starting from the index date. The rationale for stopping therapy (discontinuation with or without change of therapy) in our program concerned primary/secondary failure (i.e., failure to respond at week 16).

In this paper, the term “ineffectiveness” refers to a lack of clinical response as assessed by the treating gastroenterologist during follow-up. This includes the persistence or worsening of patient-reported symptoms, absence of meaningful clinical improvement by week 16 of therapy (primary failure), or a loss of efficacy over time following an initial positive response (secondary failure).

### Data collection

Demographic and clinical data were collected for each patient: age, sex, diagnosis, smoking, biological or biosimilar agents, prescription of other non-biological drugs, interruption or switch to another agent for a specific reason, significant comorbidity, possible primary or secondary therapeutic failure, and occurrence of AEs. AEs were classified using Medical Dictionary for Regulatory Activity (MedDRA) terminology. Following international pharmacovigilance standards, we distinguished between AEs and SAEs. AEs refer to any untoward medical occurrence in a patient during treatment with a biological drug, regardless of a causal relationship with the therapy. SAEs, on the other hand, are events that result in death, are life-threatening, require inpatient hospitalization or prolongation of existing hospitalization, result in persistent or significant disability/incapacity, or cause a congenital anomaly/birth defect [14]. In the case of the SAEs (for example, life-threatening), a switch to another biological drug was requested.

### Data processing

A descriptive analysis was conducted on data from patients admitted to the two gastroenterology units of “Renato-Dulbecco” university hospital company (Pugliese-Ciaccio Hospital of Catanzaro) and “San Giovanni di Dio” Hospital (Crotone) to assess demographic characteristics, AEs, switches and swaps of biological drug therapy. Data has been collected from 2023 to 2024. All AEs were classified according to MedDRA^®^ System Organ Class (SOC) and Preferred Term (PT) levels. The AEs have been grouped within their corresponding SOC, with PT synonyms for the same clinical condition consolidated under a single term (Table 7). All data are presented as numbers, means, or percentages. The sample size was determined by consecutive enrollment of patients admitted to both gastroenterology units during the study period. The sample size was not established in advance, and participants were recruited in consecutive order rather than through a random selection process.

## Results

The study involved a total of 214 patients enrolled between 2 November 2023 and 1 November 2024, of which 142 were recruited from the Gastroenterology Operating Unit (O.U.) of the university hospital company “Renato-Dulbecco” (Pugliese-Ciaccio) in Catanzaro, and 72 patients from the U.O. of Gastroenterology of the civil hospital “San Giovanni di Dio” in Crotone. Regarding sex distribution, males outnumbered females, as shown in Tables 1 and 2. UC was more prevalent among males (m = 90, f = 39), whereas CD was more common in females (m = 30, f = 55). Several comorbidities, especially extraintestinal manifestations, were observed across all age groups.

**Table 1:** Clinical and demographic characteristics of patients of Catanzaro hospital stratified by age, disease subtype, and comorbidity profile

Age	< 35	35–49	50–64	> 65	Naive	CD	UC
Number	42	42	31	27	71	55	87
Male (n)	31	21	14	20	45	30	56
Female (n)	11	21	17	7	26	25	31
Naïve (n)	26	20	12	13	/	28	43
CD (n)	21	18	10	6	28	/	/
UC (n)	21	24	21	21	43	/	/
Comorbidities (n)	33	36	28	25	61	49	73
Naïve comorbidities (n)	21	17	10	13	61	24	38
Cardiovascular disease (n)	0	6	9	9	8	8	16
Hypertension (n)	0	3	3	7	6	3	10
Endocrine disorders (n)	5	1	6	6	9	8	10
Osteoporosis (n)	0	0	1	6	3	2	5
Fibromyalgia (n)	2	1	1	1	4	2	3
Extraintestinal manifestation (n)	29	35	24	22	45	48	62

Data collected as part of the retrospective, observational real-world pharmacovigilance study conducted in the Calabria region between November 2023 and November 2024. Information was obtained from medical records, and follow-up evaluations were carried out by clinical pharmacologists. The table presents the characteristics of the patients included in the study and treated at the Gastroenterology O.U. of the university hospital company “Renato-Dulbecco” (Pugliese-Ciaccio) in Catanzaro. Descriptive analysis only; no statistical tests were applied. Values are expressed as the number of patients (n); total sample size: 142 patients. Abbreviation: CD = Crohn’s disease; UC = ulcerative colitis; pts = patients; n = number.

**Table 2:** Clinical and demographic characteristics of patients of Crotone hospital stratified by age, disease subtype, and comorbidity profile

Age	< 35	35–49	50–64	> 65	Naive	CD	UC
Number	27	17	16	12	38	30	42
Male (n)	12	8	7	7	22	0	34
Female (n)	15	9	9	5	16	30	8
Naïve (n)	15	9	9	5	/	10	28
CD (n)	13	6	7	4	10	/	/
UC (n)	14	11	9	8	28	/	/
Comorbidities (n)	4	5	2	3	8	10	13
Naïve Comorbidities (n)	4	1	1	2	8	3	5
Cardiovascular disease (n)	1	4	0	4	3	4	5
Hypertension (n)	1	2	3	3	2	4	5
Endocrine disorders (n)	0	0	0	0	0	0	0
Osteoporosis (n)	0	0	0	1	0	0	1
Fibromyalgia (n)	0	0	0	0	0	0	0
Extraintestinal manifestation (n)	4	6	2	4	8	6	10

Data collected as part of the retrospective, observational real-world pharmacovigilance study conducted in the Calabria region between November 2023 and November 2024. Information was obtained from medical records and follow-up evaluations carried out by clinical pharmacologists. The table presents the characteristics of patients included in the study and treated at the Gastroenterology Operating Unit (O.U.) of the civil hospital “San Giovanni di Dio” in Crotone. Descriptive analysis only; no statistical tests were applied. Values are expressed as the number of patients (n); total sample size: 72 patients. Abbreviation: CD = Crohn’s disease; UC = ulcerative colitis; pts = patients; n = number.

The average age of patients from the Gastroenterology O.U. of Catanzaro and Crotone was 53.58 and 50.3 years, respectively (Tables 3 and 4), with minimal variation compared to previous reports [15]. Regarding AEs, 69 (48.6%) were reported in Catanzaro hospital and 27 (37.5%) in Crotone hospital. No SAEs were documented in either center. Smokers accounted for 33% of patients in Catanzaro hospital and 13% in Crotone hospital.

VDZ was the most prescribed biological drug, accounting for 28.1% of prescriptions in Catanzaro and 22.2% in Crotone, reaching 50.3% of total prescriptions across both centers. UST, which was prescribed in 21.1% of patients in Catanzaro and 12.5% in Crotone, accounting for 33.6% of total prescriptions.

Among biosimilars, ADA was most prescribed in Catanzaro (56.4%), while IFX was most prescribed in Crotone (26.4%). Considering total biosimilar administration across both centers, IFX accounted for 70% and ADA for 63.3%.

The most commonly co-prescribed medications were CCSs and MSZ.

CCSs were prescribed in 53.6% of patients in Catanzaro and 47.2% in Crotone, while MSZ was used in 42.2% of patients in Catanzaro and 38.8% in Crotone (Tables 3 and 4).

**Table 3:** Patient demographics, treatment, and AEs characteristics of the Catanzaro hospital study cohort

**Overall, patients** (***n*** **= 142**)	
**Age, years**	
*Mean (± SD)*	53.58 ± 14.92
*Range (minimum-maximum)*	(min 20 - max 84)
*Median age (IQ range)*	52 (38–64)
**Gender**	
Male, n (%)	86 (60.6)
Female, n (%)	56 (39.4)
***Mean age first biologic therapy***,*** years (± SD)***	
	45.2 ± 17.6
***Naïve***,*** n (%)***	
	71 (50)
Diagnosis	
Smoking	
Smoker, n (%)	33 (23.2)
Ex-smoker, n (%)	17 (11.9)
Non-smoker, n (%)	92 (64.7)
**Prescribed biologic drugs**	
IFX, n (%)	5 (3.5)
ADA, n (%)	4 (2.8)
GOL, n (%)	7 (4.9)
UST, n (%)	30 (21.1)
UPA, n (%)	1 (0.7)
VDZ, n (%)	40 (28.1)
**Biosimilars**,** n (%)**	55 (38.7)
ADA, n (%)	31 (56.4)
IFX, n (%)	24 (43.6)
**Concurrent prescription of non-biologic drugs**	
MSZ n (%)	41 (42.2)
MTX, n (%)	1 (1.03)
CCSs, n (%)	52 (53.6)
AZP, n (%)	3 (3.09)
Switched, n (%)	10 (7.04)
Switch, n (%)	0 (0)
Swap, n (%)	10 (100)
**Adverse events**	
AEs, n (%)	69 (48.6)
SAEs, n (%)	0 (0)
Inefficacy, n (%)	10 (7.04)

Data collected as part of the retrospective, observational real-world pharmacovigilance study conducted in the Calabria region between November 2023 and November 2024. Information was obtained from medical records and follow-up evaluations carried out by clinical pharmacologists. The table presents the characteristics of patients included in the study and treated at the Gastroenterology O.U. of the university hospital company “Renato-Dulbecco” (Pugliese-Ciaccio) in Catanzaro. Results are expressed as mean ± standard deviation (SD), or number with percentage reported in parentheses, or range (minimum-maximum), or median with IQ range. Total sample size: 142 patients. Descriptive analysis only; no statistical tests were applied. Abbreviations: IFX = infliximab; ADA = adalimumab; GOL = golimumab; UST = ustekinumab; UPA = upadacitinib; VDZ = vedolizumab; MSZ = mesalazine; MTX = methotrexate; AZP = azathioprine; CCSs = corticosteroids; AEs = Adverse Events; SAEs = Serious Adverse Events; n = number; IQ = interquartile.

**Table 4:** Patient demographics, treatment, and AEs characteristics of the Crotone hospital study cohort

**Overall, patients** (***n*** **= 72**)	
**Age, years**	
*Mean (± SD)*	45.6 ± 16.2
*Range (minimum - maximum)*	(min 19 - max 80)
*Median age (IQ range)*	44 (29–61)
**Gender**	
Male, n (%)	34 (47.2)
Female, n (%)	38 (52.8)
***Mean age first biologic therapy***,*** years (± SD)***	
	38.6 ± 16.2
***Naïve***,*** n (%)***	
	38 (52.8)
**Diagnosis**	
Smoking	
Smoker, n (%)	13 (18)
Ex-smoker, n (%)	11 (15.3)
Non-smoker, n (%)	48 (66.7)
**Prescribed biologic drugs**	
IFX, n (%)	7 (9.7)
ADA, n (%)	7 (9.7)
GOL, n (%)	4 (5.5)
UST, n (%)	9 (12.5)
TFC, n (%)	4 (5.5)
UPA, n (%)	1 (1.3)
VDZ, n (%)	16 (22.2)
**Biosimilars**,** n (%)**	24 (33.3)
ADA, n (%)	5 (6.9)
IFX, n (%)	19 (26.4)
**Concurrent prescription of non-biologic drugs**	
MSZ n (%)	28 (38.8)
MP n (%)	1 (1.3)
CCSs, n (%)	34 (47.2)
AZP, n (%)	5 (6.9)
SLZ n (%)	4 (5.5)
Switched, n (%)	2 (2.8)
Switch, n (%)	0 (0)
Swap, n (%)	2 (100)
**Adverse events**	
AEs, n (%)	27 (37.5)
SAEs, n (%)	0 (0)
Inefficacy, n (%)	2 (2.8)

Data collected as part of the retrospective, observational real-world pharmacovigilance study conducted in the Calabria region between November 2023 and November 2024. Information was obtained from medical records and follow-up evaluations carried out by clinical pharmacologists. The table presents the characteristics of patients included in the study and treated at the Gastroenterology Operating Unit (O.U.) of the civil hospital “San Giovanni di Dio” in Crotone. Results are expressed as mean ± standard deviation (SD), or number with percentage reported in parentheses, or rage (minimum-maximum), or median with IQ range. Total sample size: 72 patients. Descriptive analysis only; no statistical tests were applied. Abbreviations: IFX = infliximab; ADA = adalimumab; GOL = golimumab; UST = ustekinumab; UPA = upadacitinib; VDZ = vedolizumab; MSZ = mesalazine; MTX = methotrexate; AZP = azathioprine; SLZ = sulfasalazine; MP = Mercaptopurine; AEs = Adverse Events; SAEs = Serious Adverse Events; n = number; IQ = interquartile.

### Switches and swaps related to inefficacy

**Fig. 1:**
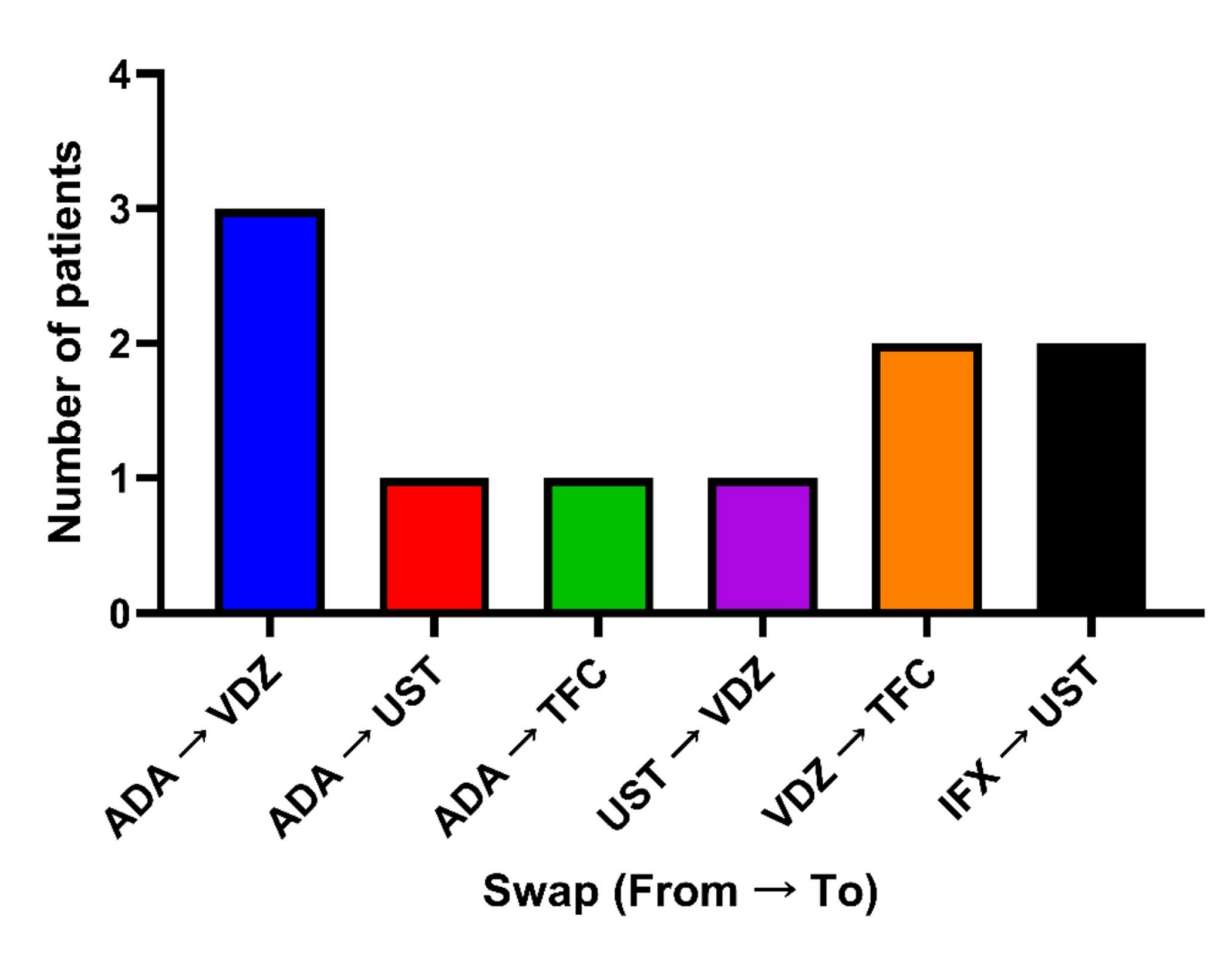
Therapy swaps between monoclonal antibodies

Twelve therapy changes were observed in our cohort, all involving swaps to different biological agents (no switches within the same drug class were recorded). All therapy swaps (Table 5) occurred due to treatment inefficacy; none were attributed to AEs. Among the twelve therapy swaps, the most frequent target therapies were UST and VDZ (33.3% each), followed by TFC (25%) and ADA (8.3%). In addition, the most frequent therapy swap was from ADA to VDZ (25% of all swaps) (Fig. 1; Table 5).

Data collected as part of the retrospective, observational real-world pharmacovigilance study conducted in the Calabria region between November 2023 and November 2024. Information was obtained from medical records and follow-up evaluations conducted by clinical pharmacologists. The data presented in this figure refer to the 12 cases of therapeutic swaps observed within the overall study population, which included 214 patients treated at the gastroenterology units of the hospitals of Catanzaro and Crotone. Descriptive analysis only; no statistical tests were applied. Abbreviations: IFX = infliximab; ADA = adalimumab; UST = ustekinumab; VDZ = vedolizumab; TFC = tofacitinib.

**Table 5:** Switches related to inefficacy

Switch/Swap to							
Switch/Swap from	IFX	ADA	GOL	UST	VDZ	TFC	UPA
IFX		-	-	0/2	-	-	-
ADA	-		-	0/1	0/3	0/1	-
GOL	-	-		-	-	-	-
UST	-	-	-		0/1	-	-
VDZ	-	0/1	-	0/1		0/2	-
TFC	-	-	-	-	-		-
UPA	-	-	-	-	-	-	

Data collected from the retrospective, observational real-world pharmacovigilance study conducted in the Calabria region between November 2023 and November 2024. Information was obtained from medical records and follow-up evaluations conducted by clinical pharmacologists. The table reports the 12 cases of therapeutic swap observed within the overall study population, which included 214 patients treated at the gastroenterology units of the hospitals of Catanzaro and Crotone. Descriptive analysis only; no statistical tests were applied. Abbreviations: IFX = infliximab; ADA = adalimumab; UST = ustekinumab; VDZ = vedolizumab; TFC = tofacitinib; GOL = golimumab; UPA = upadacitinib.

### Descriptions of AEs

During the study period, we observed 96 (44.8%) cases of AEs and 0 (0%) cases of SAEs. VDZ was associated with the highest number of AEs, accounting for 31 cases (32.3%), followed by IFX with 22 cases (23.0%), ADA and UST with 17 cases each (17.7%), and GOL with 7 cases (7.3%). UPA and TFC had the fewest AEs, with one case each (*n* = 1; 1.0%) (Table 6).

Table 7 shows an extensive list of AEs and SAEs categorized according to the MedDRA dictionary. The most common AEs were GI disorders (*n* = 24; 25.0%) with symptoms such as diarrhea and abdominal pain, followed by general disorders (asthenia and pyrexia) or administration site conditions (*n* = 22; 23%), and skin and subcutaneous tissue disorders (*n* = 16; 16.7%) with pruritus and rash reactions (Table 7).

**Fig. 2:**
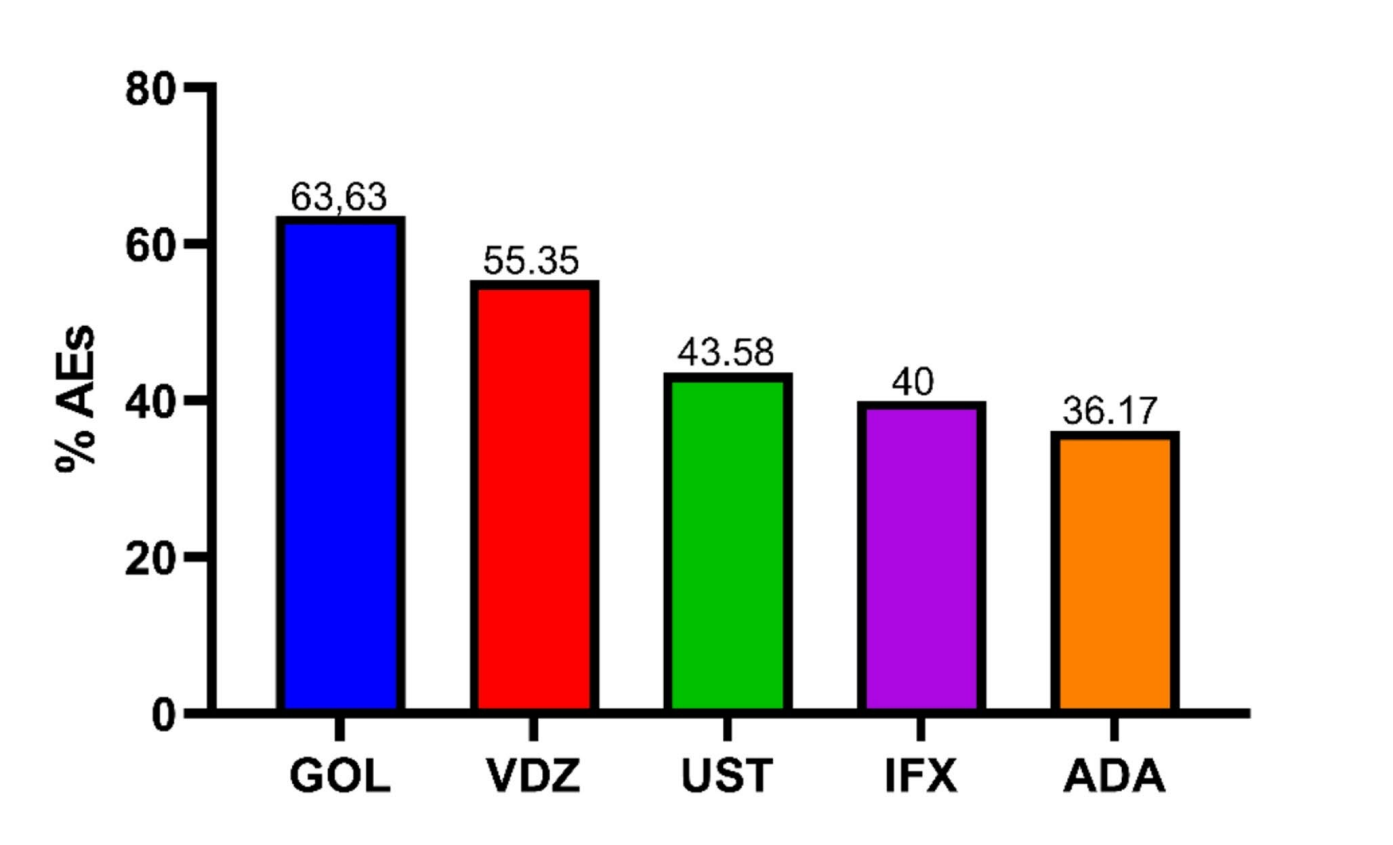
Percentage of reported AEs for Each Monoclonal Antibody

The analysis of biological drug substitutions due to loss of efficacy, observed in 12 patients, indicates a trend toward replacing TNF-α inhibitors with therapies that target specific pathways. These include treatments aimed at interleukins, Janus kinases, and the α4β7 integrin (Fig. 2; Table 7).

Data collected from the retrospective, observational real-world pharmacovigilance study conducted in the Calabria region between November 2023 and November 2024. Information was obtained from medical records and follow-up evaluations conducted by clinical pharmacologists. The graph represents the AEs observed within the overall study population, which included 214 patients treated between the gastroenterology units of the hospitals of Catanzaro and Crotone. The data is as follows:

GOL: 7 AEs out of 11 prescriptions; VDZ: 31 AEs out of 56 prescriptions; UST: 17 AEs out of 39 prescriptions; IFX: 22 AEs out of 55 prescriptions; ADA: 17 AEs out of 47 prescriptions. Only drugs prescribed to more than 10 patients are reported. Therefore, TFC (1 AEs/4 prescriptions) and UPA (1 AEs/2 prescriptions) are excluded from the graph because of the limited number of prescriptions. Data are as a percentage of reported AEs in relation to the total number of prescriptions for each drug; descriptive analysis only; no statistical tests were applied. Abbreviations: IFX = infliximab; ADA = adalimumab; UST = ustekinumab; VDZ = vedolizumab; GOL = golimumab; AEs = adverse events.

**Table 6:** Characteristics of the study cohort of patients affected by IBDs treated with biological and non-biological agents

Patients number		ADA	GOL	IFX	TFC	UPA	UST	VDZ
	214	47	11	55	4	2	39	56
Mean age (± SD)	48.3 ± 14.7	45.8 ± 15.2	47.5 ± 13.3	43.6 ± 15.7	53.2 ± 16.5	45.5 ± 0.5	46.1 ± 15.2	50.3 ± 18.1
Range (minimum - maximum)	19–84	22–83	20–56	19–81	29–74	45–46	22–84	21–83
Median age (IQ range)	48 (36–60)	46 (34–58)	47 (38–58)	43 (30–57)	55 (41–65.5)	45.5 (-)	45 (33–58)	50 (36–64)
Female, n (%)	94 (43.9)	24 (25.5)	4 (4.2)	27 (28.7)	3 (3.2)	1 (1.06)	18 (19.1)	17 (18.1)
Male, n (%)	120 (56.1)	23 (19.2)	7 (5.8)	28 (23.3)	1 (0.8)	1 (0.8)	21 (17.5)	39 (32.5)
UC, n (%)	129 (60.3)	20 (15.5)	8 (6.2)	37 (28.7)	2 (50)	1 (1,5)	14 (10.8)	47 (36.4)
CD, n (%)	85 (39.7)	27 (31.8)	3 (3.5)	18 (21.2)	2 (2.35)	1 (1.2)	25 (29.4)	9 (10.6)
MTX, n (%)	1 (0.5)	-	-	-	-	-	1 (100)	-
MSZ, n (%)	69 (32.2)	13 (18.8)	4 (5.8)	19 (27.5)	1 (1.44)	1 (1.44)	11 (15.9)	20 (29.0)
CCSs, n (%)	86 (40.2)	16 (18.6)	5 (5.8)	23 (26.7)	3 (3.5)	1 (1.2)	17 (19.8)	21 (24.4)
SLZ, n (%)	4 (1.9)	1 (25)		1 (25)	-	-	1 (25)	1 (25)
AZT, n (%)	8 (3.7)	3 (37.5)	2 (25.0)	1 (12.5)	-	-	2 (25.0)	-
MP, n (%)	1 (0.5)	-	-	-	-	-	1 (100)	-
Switch, n (%)	-	-	-	-	-	-	-	-
Swap, n (%)	12 (5.6)	5 (41.7)		2 (16.7)	-	-	1 (8.3)	4 (33.3)
AEs, n (%)	96 (44.8)	17 (17.7)	7 (7.3)	22 (23.0)	1 (1.0)	1 (1.0)	17 (17.7)	31 (32.3)
SAEs, n (%)	-	-	-	-	-	-	-	-

Data originated from the retrospective, observational real-world pharmacovigilance study conducted in the Calabria region between November 2023 and November 2024. Information was obtained from medical records and follow-up evaluations conducted by clinical pharmacologists. The table reports the demographic and clinical characteristics of patients, the therapies undertaken with both biologic and non-biologic agents, and the occurrence of AEs or SAEs. Data were obtained by the overall study population, which included 214 patients treated in the gastroenterology units of the hospitals of Catanzaro and Crotone. Results are expressed as the number of patients with percentage reported in parentheses, or as number with SD, or as range (minimum-maximum), or as IQ range.

Descriptive analysis only; no statistical tests were applied. Abbreviations: IFX = infliximab; ADA = adalimumab; UST = ustekinumab; VDZ = vedolizumab; GOL = golimumab; TFC = tofacitinib; UPA = upadacitinib; MP = Mercaptopurine; CD = Crohn’s disease; UC = ulcerative colitis; SD = standard deviation; IQ = interquartile; n = number; MTX = methotrexate; MSZ = mesalazine; CCSs = corticosteroids; SLZ = sulfasalazine; AZT = azathioprine; AEs = Adverse Events; SAEs = Serious Adverse Events.

**Table 7:** MedDRA-compliant description of AEs

MedDRA-compliant description of AEs	ADA	GOL	IFX	TFC	UPA	UST	VDZ	Total
**SOC - General disorders and administration site conditions**	3	2	5		1	4	7	22
PT - Lack of appetite						1	1	2
PT1 – Asthenia	2		2			2	2	8
PT2 - Hot flush								-
PT3 – Drowsiness			1					1
PT4 - Administration site reactions			1			1	1	3
PT5 - Peripheral edema							1	1
PT6- Pyrexia	1	2	1		1		2	7
**SOC - Vascular disorders**	**1**					**1**	**5**	**7**
PT – Cyanosis								-
PT1 - Hypotension							1	1
PT2 - Tachycardia	1					1	4	6
**SOC - Skin and subcutaneous tissue disorders**	**1**		**6**			**4**	**5**	**16**
PT – Folliculitis								-
PT1 – Rash							1	1
PT2 – Pruritus	1		6			4	4	15
PT3- Alopecia								-
PT4-Tingling feet/hands								-
**SOC - Ear and labyrinth disorders**								**-**
PT – Vertigo								**-**
**SOC - Nervous system disorders**	**1**	**1**	**1**			**1**		**4**
PT – Headache	1	1	1			1		4
**SOC - Infections and infestations**	**1**					**1**	**3**	**5**
PT - Candidiasis infection								**-**
PT1 - Herpes virus infection	1					1	2	4
PT2 - Tuberculosis flare-up							1	1
**SOC - Respiratory**,** thoracic**,** and mediastinal disorders**			**1**			**3**	**2**	**6**
PT - Pneumonia								**-**
PT1 - Interstitial pneumonia								**-**
PT2 - Nasopharyngitis								**-**
PT3 - Bronchospasm			1			3	2	6
PT4 - Dyspnoea								-
**SOC - Investigations**	**2**			**1**				**3**
PT - Transaminases increased	1			1				2
PT1 - Hepatitis C antibody positive								-
PT2 - Carcinoembryonic antigen increased								-
PT3 - Blood count abnormal								-
PT4 - Haemoglobin decreased								-
PT5 - Red blood cell sedimentation rate abnormal	1							1
**SOC - Blood and lymphatic system disorders**	**1**						**1**	**2**
PT - Lymphocytosis							1	1
PT1 - Thrombocytopenia								-
PT2 – Anaemia	1							1
PT3 - Splenomegaly								-
**SOC - Gastrointestinal disorders**	**5**	**4**	**6**			**2**	**7**	**24**
PT – Vomiting	2	1						3
PT1 - Dyspepsia			1			1		2
PT2 – Nausea								-
PT3 - Diarrhea	2	1					4	7
PT4 - Rectorrhagia	1	1	1					3
PT5 - Abdominal pain		1	4			1	3	9
**SOC - Immune system disorders**								**-**
PT - Allergic reaction to excipient								**-**
PT1 - Lupus-like syndrome								**-**
**SOC - Renal and urinary disorders**								**-**
PT - Haematuria								**-**
PT1 - Haemorrhagic Cystitis								**-**
**SOC – Musculoskeletal and connective tissue disorders**	**2**		**3**			**1**	**1**	**7**
PT - Limb discomfort	2		2			1	1	6
PT1 – Myalgia			1					1
**Total AEs**	**17**	**7**	**22**	**1**	**1**	**17**	**31**	**96**

Data originated from the retrospective, observational real-world pharmacovigilance study conducted in the Calabria region between November 2023 and November 2024. Information was obtained from medical records and follow-up evaluations conducted by clinical pharmacologists. The table reports the description of AEs. Data were obtained by the overall study population, which included 214 patients treated in the gastroenterology units of the hospitals of Catanzaro and Crotone. Results are expressed as the number of AEs recorded. Descriptive analysis only; no statistical tests were applied. Abbreviations: MedDRA = Medical dictionary for regulatory activity; SOC = System organ class; PT = preferred term; IFX = infliximab; ADA = adalimumab; UST = ustekinumab; VDZ = vedolizumab; GOL = golimumab; UPA = upadacitinib; AEs = Adverse Events.

## Discussion

In the present retrospective and observational study conducted in two gastroenterology centers in Calabria, Italy, 214 patients with IBDs treated with biologic drugs between 2023 and 2024 were enrolled. The results show that VDZ was the most prescribed biologic (50.3%), followed by UST (33.6%), while IFX and ADA were the most used biosimilars. A total of 96 AEs (44.8%) were recorded, none of which were classified as serious. The most common AEs were GI complaints, general conditions, and skin reactions. Furthermore, all 12 therapy changes (swaps) were made due to clinical ineffectiveness, not AEs. These data underscore the importance of pharmacovigilance in monitoring the safety and efficacy of biologics in a real-world setting, contributing to more targeted and personalized therapeutic choices.

The start of clinical therapy using biologics marks a milestone in modern medicine. Over the past two decades, thanks to their value and safety, these agents have revolutionized the management of numerous immune-mediated diseases, such as IBDs [16]. The history of monoclonals in gastroenterology began with the discovery of anti-TNF-α biologics. In particular, IFX was the first anti-TNF-α chimeric mAb used in the treatment of CD and whose use was later extended to other therapeutic areas, including UC, paediatric CD, and rheumatological diseases [17].

A further and highly significant improvement in the pharmacology of mAbs was achieved with the production of humanized anti-TNF-α mAbs such as ADA and GOL, bringing the great advantage of reduced immunogenicity while still maintaining certain limitations [17, 18].

The use of anti-TNF biologics is well established in clinical practice, as they have been the mainstay of treatment of moderate to severe IBDs for over 20 years [19]. “As expected, anti-TNF therapy was the most frequently prescribed treatment in our study population when considering the prescription rates of biosimilar drugs in the two operating units analyzed. However, when focusing solely on the prescription of originator biologics, both the Gastroenterology Units of Catanzaro and Crotone showed a notable increase in the use of biologics targeting α4β7 integrin and interleukins (VDZ and UST, respectively). Indeed, VDZ has demonstrated efficacy, especially in moderate to severe UC, becoming a good candidate for first-line biological therapy [15, 20]. Although many medical society guidelines do not provide specific recommendations, current evidence-based management algorithms generally reserve UST for second-line therapy. Therefore, we can affirm that the prescribing trend defined in this study aligns with the scientific evidence and recommendations available in the literature [20]. Kapizioni et al. reported that VDZ is more effective when used as a first-line therapy compared to later lines. Additionally, their study identified 1.168 patients who were refractory to one or two anti-TNF biological therapies but subsequently responded well to VDZ or UST as second- or third-line treatments. They also demonstrated that these two biologics exhibited similar efficacy over a 3-year follow-up period [21].

In our one-year study, the occurrence of AEs in IBD patients during biologic therapy amounted to a total of 96 AEs (44.8%), consistent with those already known and reported in different published studies [22].

The most frequently described AEs during the study in our patients were GI disorders (diarrhea, abdominal pain) and some general disorders such as asthenia and pyrexia.

Several pharmacovigilance studies, in agreement with our results, have already shown that the most frequently encountered AEs include those we report here [23, 24].

Although the immunomodulatory action of biological drugs may predispose patients to an increased susceptibility to infections, we did not report severe infections in our study.

Data from a recent meta-analysis showed that VDZ and UST were associated with a reduced risk of infection [25]. Since these two drugs were the most prescribed biologics in our patient cohort, the absence of infectious side effects in our study is not surprising and is consistent with existing literature data.

The adoption of new JAK kinase inhibitor drugs, such as TFC and UPC, broadened the therapeutic options, helping to improve the clinical management of patients [26].

As documented in other studies, the absence or loss of efficacy remains the primary cause for changing biological therapy [27, 28]. In our study, all swaps performed were motivated by therapeutic inefficacy. Of the twelve therapy exchanges identified, those most frequently recorded concerned the change to a new therapy with UST, VDZ (33.3% for both), or TFC (25%). In our study, 5 out of 12 therapy swaps were due to ADA ineffectiveness. This finding agrees with another recent study in which 301 patients with UC received ADA as the first biological agent and, after therapy failure, continued with IFX or VDZ. VDZ has previously demonstrated significantly better treatment survival without interruption or failure compared to ADA, with up to 2 years of follow-up [21]. In addition, the most frequent therapy exchange was from ADA to VDZ (25%). This is justified by the fact that patients who experience therapeutic failure with anti-TNF drugs are often switched to therapies with different mechanisms of action [29].

Indeed, the VARSITY clinical trial conducted a few years ago in patients with moderate to severe active ulcerative colitis had already demonstrated the superiority of VDZ over ADA in terms of achieving clinical remission and endoscopic improvement in UC [30].

For example, VDZ is a selective α4β7 integrin inhibitor that also presents the advantage of having an intestine-specific effect, thereby reducing the risk of systemic immunosuppression [31]; UST which is an IL12/23 inhibitor has a favorable efficacy and tolerability profile, especially in CD [32]; and TFC, a JAK inhibitor indicated especially in UC, is useful in treating patients who are refractory or non-responsive to biologics [33].

Moreover, the treat-to-target approach, in which more emphasis is placed on the personalization of therapy, is becoming increasingly popular, leading clinicians to prefer drugs targeted according to the patient’s response and pharmacological history.

This study has some limitations. First, it is an observational study without a control group, which limits the ability to establish causal relationships. Second, the follow-up period was relatively short (12 months), which may not fully capture long-term safety signals or treatment outcomes. Third, while AEs were thoroughly reported, the study did not include patient-reported outcomes such as quality of life (QoL), disease activity indices, or endoscopic findings. These parameters would provide a more comprehensive view of treatment effectiveness from a patient-centered perspective. Future studies with longer duration, inclusion of comparator arms, and patient-reported outcomes are warranted to strengthen the evidence.

## Conclusions

Given the widespread use of biological drugs in IBDs, pharmacovigilance activities play a crucial role in improving the detection and monitoring of AEs in clinical practice. Compared to previously published data from the same centers (2017–2018), we observed a decrease in reported AEs rates and the absence of SAEs.

Despite the relatively short study duration, our findings provide valuable real-world data on biological drug usage, safety, and efficacy in gastroenterology practice. The observed decrease in AEs compared to historical data suggests improved safety outcomes, though the underlying factors contributing to this improvement require further investigation.

Treatment inefficacy remains a primary concern, as all therapy changes in our cohort were attributed to a lack of efficacy rather than AEs. Enhanced reporting of drug-related inefficacy could provide valuable insights for optimizing patient selection and reducing unnecessary therapy switches. This observational study has some limitations, including the relatively small sample size, short follow-up period, potential selection bias, and inability to control for confounding variables. Therefore, causal relationships between patient characteristics and specific AEs cannot be established. Future prospective studies with larger sample sizes, longer follow-up periods, and control groups are needed to provide more robust evidence regarding the safety and efficacy of biological therapies in IBD patients.

## Data Availability

No datasets were generated or analysed during the current study outside of those already reported in the text.

## Abbreviations

ADA: Adalimumab
AEs: Adverse Events
AIFA: Italian Medicines Agency
AZT: Azathioprine
CBPP: Calabria Biologics Pharmacovigilance Program
CCSs: Corticosteroids
CD: Crohn’s Disease
GI: Gastrointestinal
GOL: Golimumab
IBDs: Inflammatory Bowel Diseases
IFX: Infliximab
IL-12/23: Interleukin-12/23
IQ: Interquartile
JAK: Janus Kinase
MedDRA: Medical Dictionary for Regulatory Activity
MP: Mercaptopurine
MSZ: Mesalazine
MTX: Methotrexate
O.U.: Operating Unit
PT: Preferred Term
PTS: Patients
QoL: Quality of Life
SAEs: Serious Adverse Events
SD: Standard Deviation
SLZ: Sulfasalazine
SOC: System Organ Class
TFC: Tofacitinib
Th1: T helper 1
Th2: T helper 2
Th17: T helper 17
TNF: Tumor Necrosis Factor
UC: Ulcerative Colitis
UPA: Upadacitinib
UST: Ustekinumab
VDZ: Vedolizumab
